# Evidence of Directional Structural Superlubricity and Lévy Flights in a van der Waals Heterostructure

**DOI:** 10.1002/smll.202408349

**Published:** 2024-11-26

**Authors:** Maxime Le Ster, Paweł Krukowski, Maciej Rogala, Paweł Dabrowski, Iaroslav Lutsyk, Klaudia Toczek, Krzysztof Podlaski, Tefvik Onur Menteş, Francesca Genuzio, Andrea Locatelli, Guang Bian, Tai‐Chang Chiang, Simon A. Brown, Paweł J. Kowalczyk

**Affiliations:** ^1^ Faculty of Physics and Applied Informatics University of Lodz Pomorska 149/153 Lodz 90‐236 Poland; ^2^ Elettra‐Sincrotrone Trieste S.C.p.A. Strada Statale 14 ‐ km 163,5 in AREA Science Park, Basovizza 34149 Trieste Italy; ^3^ Department of Physics and Astronomy University of Missouri Columbia MO 65211 United States; ^4^ Department of Physics University of Illinois at Urbana‐Champaign Urbana IL 61801‐3080 United States; ^5^ The MacDiarmid Institute for Advanced Materials and Nanotechnology University of Canterbury Christchurch 8140 New Zealand

**Keywords:** alpha‐bismuthene, LEEM, Lévy flights, nanohighways, structural superlubricity

## Abstract

Structural superlubricity is a special frictionless contact in which two crystals are in incommensurate arrangement such that relative in‐plane translation is associated with vanishing energy barrier crossing. So far, it has been realized in multilayer graphene and other van der Waals (2D crystals with hexagonal or triangular crystalline symmetries, leading to isotropic frictionless contacts. Directional structural superlubricity, to date unrealized in 2D systems, is possible when the reciprocal lattices of the two crystals coincide in one direction only. Here, directional structural superlubricity a α‐bismuthene/graphite van der Waals system is evidenced, manifested by spontaneous hopping of the islands over hundreds of nanometers at room temperature, resolved by low‐energy electron microscopy and supported by registry simulations. Statistical analysis of individual and collective α‐bismuthene islands populations reveal a heavy‐tailed distribution of the hopping lengths and sticking times indicative of Lévy flight dynamics, largely unobserved in condensed‐matter systems.

## Graphical abstract

In‐situ Low Energy Electron Microscopy sequences of α‐bismuthene islands grown on highly‐ordered pyrolithic graphite reveal spontaneous directional hopping behavior promoted by directional structural superlubricity, to date unrealized in van der Waals heterostructures. Statistical analysis of the hopping lengths and sticking times of the α‐Bi islands are consistent with Lévy flight dynamics, which has been largely unobserved in condensed‐matter systems.

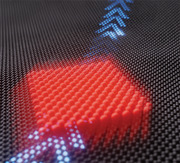

## 1 Introduction

Friction emerges from energy dissipation at the contact interface between two materials in relative motion and is present in virtually all mechanical systems. Friction, responsible for both direct energy losses (essentially, heat dissipation) and indirect costs (performance reduction, material wear and repair) has been estimated to contribute to approximately 23% of the world's energy consumption.^[^ ^1^, ^2^ ^]^ Therefore, it is desirable to investigate interfacial systems that offer a substantial decrease in friction coefficients. Structural superlubricity^[^ ^3^, ^4^, ^5^, ^6^, ^7^, ^8^ ^]^ is a regime of motion between crystalline solids in incommensurate contact (with no additional lubricant phase) leading to considerable friction coefficient reduction typically below instrumental resolution.^[^ ^3^, ^4^ ^]^ Over the last decade, structural superlubricity has been described in a number of van der Waals (vdW) 2D systems such as graphene on graphite,^[^ ^3^, ^9^ ^]^ bilayer graphene,^[^ ^10^ ^]^ multilayer hexagonal boron nitride (hBN)^[^ ^3^ ^]^ and molybdenum disulfide (MoS_2_) on graphite and hBN.^[^ ^11^ ^]^ Despite a considerable progress in the investigation of superlubricity in 2D vdW systems, the reported component crystalline layers almost always possess a triangular or hexagonal surface symmetry (graphene, MoS_2_, hBN). To the best of our knowledge, the only exception is a theoretical investigation of bilayer α‐phosphorene;^[^ ^12^ ^]^ however, no nanoscale systems involving superlubricity with distinct symmetries have been published so far.

In recent years, phenomenological models were developed by Hod^[^ ^3^, ^4^ ^]^ which showed that atomic registry at the crystalline interface is crucial to understanding structural superlubricity. To that effect, registry index (RI) simulations, which track the atomic overlap at the interface, offer qualitative approximations of the translational energy landscape *U*(**r**_*C*_) (with **r**_*C*_ the relative translation vector). Recently, Panizon^[^ ^13^ ^]^ classified three types of crystalline contacts which depend on the set of coincidence reciprocal lattice vectors Ω at the crystalline interface (Ω can be either empty, or form a 1D, or a 2D lattice). The type of contact determines the structure of *U*(**r**_*C*_) which can be corrugated in all directions (type‐A), in one direction only (type‐B), or uniform (type‐C). In type‐A contacts, a translation of the adsorbate layer requires overcoming non‐zero energy barriers in all translation directions; this regime of motion corresponds to *directional locking*,^[^ ^14^ ^]^ prohibiting structural superlubricity. Type‐C contacts on the contrary lead to vanished energy barriers in all translation directions, *i.e*., structural superlubricity as described in most experimental reports.^[^ ^3^, ^4^, ^5^, ^6^, ^7^, ^9^, ^10^, ^11^ ^]^ Lastly, type‐B contacts lead to 1D translation energy landscapes such that the adsorbate layer can glide freely along directional tracks (*nanohighways*) associated with quasi‐vanishing energy barriers, while other directions lead to much larger friction coefficients, typical of type‐A. Type‐B is possible only when the two lattices have different rotational symmetries,^[^ ^13^ ^]^ and was demonstrated experimentally in a self‐assembled triangular lattice of colloidal particles on a surface with square symmetry (with lattice parameters of the order of several micrometers).^[^ ^13^, ^15^ ^]^ However, there is to date no evidence of such directional structural superlubricity regime in a nanoscale system with atomically‐clean contact.

In this work, we report the first realization of directional structural superlubricity in a nanoscale 2D system, comprised of self‐assembled α‐bismuthene (α‐Bi) islands (i.e., atomically‐thin and atomically smooth nanoparticles) on a highly‐ordered pyrolithic graphite (HOPG) substrate. Ultra‐thin films of α‐Bi have been synthesized on HOPG^[^ ^16^, ^17^, ^18^, ^19^, ^20^, ^21^, ^22^, ^23^, ^24^, ^25^, ^26^ ^]^ and on other substrates.^[^ ^27^, ^28^, ^29^, ^30^, ^31^, ^32^, ^33^, ^34^, ^35^, ^36^, ^37^, ^38^ ^]^ X‐ray photoelectron spectroscopy (XPS),^[^ ^18^ ^]^ angle‐resolved XPS (ARPES)^[^ ^39^ ^]^ and scanning tunneling microscopy (STM)^[^ ^23^ ^]^ reveal fully saturated Bi bonds and a semi‐metallic bandstructure indicating that the α‐Bi/HOPG interface is of the van der Waals type, as further evidenced by the presence of moiré patterns.^[^ ^24^, ^25^ ^]^ While α‐Bi has been the subject of numerous studies investigating its electronic and topological properties,^[^ ^21^, ^23^, ^39^, ^40^ ^]^ little is known about its mechanical properties, in particular regarding superlubricity. In this paper, we use low‐energy electron microscopy (LEEM) during and after Bi deposition to observe, in real time (at a frame rate up to 0.24 s) and real space, the motion dynamics of α‐Bi islands, which appear to spontaneously hop back‐and‐forth (along graphitic zigzag directions) with hopping lengths ℓ as large as 600 nm. Our RI simulations support these observations and reveal a unique translational energy potential landscape as a function of the twist angle θ between α‐Bi and HOPG, in agreement with the predicted directional superlubricity which enables spontaneous diffusion of the islands at room temperature. Interestingly, the distributions of hopping lengths and sticking times (duration between successive hopping events) *P*(ℓ) and *P*(τ) respectively, for both individual islands and for the global population are heavy‐tailed (i.e., which decays slower than exponentially; *P*(ℓ) ∼ ℓ^−2.2^ and *P*(τ) ∼ τ^−2.3^), highlighting that α‐Bi islands on graphite possess Lévy flights dynamics, which have been extremely scarce in solid‐state physics, *a fortiori* for large and massive nanostructures comprised of up to hundreds of thousands of atoms.

## 2 LEEM and µ‐LEED Results

### 2.1 General Description of the Growth

**Figure 1:**
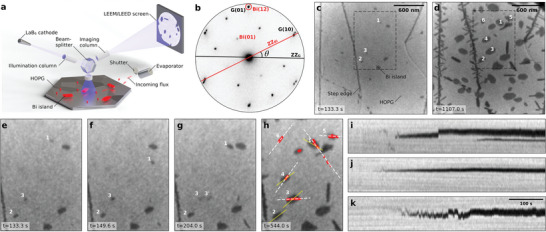
LEEM and µ‐LEED experiments. a) Schematics of the experimental setup. b) µ‐LEED pattern (*E* = 40 eV) of a single α‐Bi island. The black and red lines indicate graphite and α‐Bi zigzag directions, respectively. The twist angle (θ ≃ 28°) is indicated. c) and d) 2.25 × 2.25~µm^2^ LEEM images recorded during Bi deposition at 133.3 and 1107.0 s, respectively. e–h) Sequence of LEEM images (area in the dashed squares in (c, d)) showing α‐Bi growth snapshots recorded at 133.3, 149.6, 204.0, and 544.0 s into deposition. The yellow lines in (h) indicate α‐Bi elongation (zigzag) direction, and the red dots indicate the position of the center of mass of the islands during deposition (dashed white lines are linear regressions). i–k) Time‐dependent cross‐sections of islands 1, 2, and 3 respectively (panel vertical dimension: 500 nm). All measurements in (b–k) were obtained at room temperature.

α‐Bi islands were grown on HOPG using a simple atomic deposition process described in depth in previous reports.^[^ ^16^, ^17^, ^18^, ^19^, ^20^, ^21^, ^22^, ^24^, ^25^, ^29^, ^39^, ^40^, ^41^ ^]^ In this work, the growth process is observed using LEEM and micro‐spot low‐energy electron diffraction (µ‐LEED).^[^ ^42^, ^43^, ^44^ ^]^ **Figure** **1**a illustrates the experimental set‐up employed to monitor in‐situ the Bi deposition on HOPG. Figure 1b shows the diffraction pattern from a single α‐Bi island, obtained by restricting the e‐beam illumination to a spot size of the order of 500 nm. The diffraction pattern resolves both α‐Bi and the underlying HOPG substrate giving an estimate of the twist angle θ ≃ 28°, in agreement with previous experimental reports.^[^ ^24^, ^25^, ^40^, ^45^, ^46^ ^]^ Interestingly, the µ‐LEED pattern shows a unique reciprocal lattice coincidence vector **G**(01) = **Bi**(12), indicating a directional commensurate matching, which is a crucial factor in terms of anisotropic diffusion.^[^ ^13^, ^15^ ^]^ In fact, by symmetry, θ = 32° should lead to a similar situation where the coincidence vector is $G\left(\bar{1}1\right)=\mathrm{Bi}\left(\bar{1}2\right)$ (see Section S1, Supporting Information). Figure 1c,d shows LEEM images obtained (c) shortly after island nucleation, i.e., at the early stages of growth, with small islands decorating graphite step edges and occasionally located in the middle of the terraces; and (d) after deposition of a Bi dose equivalent to a coverage of ∼1  monolayer (ML). The LEEM image shown in Figure 1d is representative of the later stages of α‐Bi growth on HOPG. Besides step‐edge decoration, one can observe two different types of α‐Bi crystals: (i) relatively large and flat islands with thickness in the range 2‐4 ML; (ii) narrower and elongated nanorods, both elongated along a preferential direction parallel to α‐Bi **R**_1_ direction analogous to bulk Bi $\left\langle 1\bar{1}0\right\rangle$ direction.^[^ ^16^, ^17^, ^18^, ^21^, ^25^, ^39^, ^40^ ^]^ Note that most islands are anchored at steps, which act as nucleation centers in the early growth stages. We occasionally observe island ripening, which is common for 2D systems in which adatom attachment/detachment takes place between the condensed island and the 2D lattice gas.^[^ ^22^ ^]^ Previous studies show that relatively small differences in the substrate temperature during growth (and post‐growth annealing)^[^ ^17^ ^]^ can lead to significant changes in the morphology of the α‐Bi islands. The absence of such changes in our LEEM images confirms that the islands remain close to room temperature.

### 2.2 Spontaneous Hopping

Quantitative analysis of LEEM movies shows that, during Bi deposition at room temperature, about ∼20% of the islands (including ones with areas as large as 20000 nm^2^) spontaneously diffuse along straight lines in a back‐and‐forth fashion, which we refer to as *hopping*. The complete LEEM sequence is shown in Movie S1 (Supporting Information) recorded during the entire deposition (about 18 min). Several zoomed‐in frames are shown in Figure 1e–h. The hopping behavior is clearly observed for island 1, captured in four different positions in all panels, in contrast to island 2 which remains stationary during deposition. The centre of mass of islands 1–6 recorded over the whole deposition are overlaid onto Figure 1h and confirm the directional character of the hopping, where the α‐Bi islands diffuse in straight lines (white dashed lines) along directions ≃ 30° off their elongation direction (solid yellow lines). Furthermore, the hopping directions of the different islands are rotated by ±60°, suggesting that this phenomenon is governed by graphitic crystallographic directions. The variations within the island population is highlighted by the waterfall plots in Figure 1i–k. Island 1 diffuses back‐and‐forth rapidly at the beginning of the deposition and then stabilizes for about 100 s, finally hopping once more and remains stationary for the rest of the deposition. In contrast, island 2 is fixed during the entire deposition, likely due to defect‐pinning.^[^ ^47^ ^]^ Island 3, on the other hand, appears to shuttle to and from two fixed locations, likely alternating between two shallow pinning states caused by local defects in the HOPG crystalline structure. Closer inspection of islands 3 and 4 in Figure 1h shows that despite having nearly parallel elongation axes (and therefore twist angles θ) their spontaneous hopping directions differ significantly (≃60°). In fact, the two islands appear to have a slightly different twist (yellow lines are not exactly parallel). This unique friction/twist dependence, in which a twist change of several degrees leads to a drastic change the in easy‐translation direction, is corroborated below with our simulations.

### 2.3 Origin of Hopping

In LEEM, the electron energy at the sample surface is very low (few tens of eV) compared to the much higher energies found in transmission electron microscopy (TEM; around 100 keV). Therefore in our case we can rule out electron‐induced processes such as cluster diffusion, previously observed in TEM studies.^[^ ^47^, ^48^ ^]^ We find no evidence that the Bi flux influences the hopping behavior of the α‐Bi islands. The very low deposition rate (∼0.01 Å s^−1^), the high thermal stability and thermal conduction of graphite, as well as the substrate being held at room temperature during the experiment rule out thermal effects from the Bi vapor during deposition. Instead, we attribute the island diffusion to surface phonons, as was observed for C_60_ molecules on Au(111) surface^[^ ^49^ ^]^ and Au clusters on graphite.^[^ ^50^ ^]^ We investigate the influence of the substrate thermal activity by increasing the temperature (∼400 K, see Section S3 and Movie S3, Supporting Information) which results in the unpinning of several islands previously immobilized at room temperature, further indicating that the momentum transfer has a thermal origin and that pinning/unpinning activation processes occur at defect sites.

### 2.4 Diffusion Velocity

Due to the limited time resolution of the LEEM set‐up at high magnifications (0.2 − 2.7 frames per second), estimating the velocity of the α‐Bi islands during a diffusion event is difficult, due to both under‐sampling and the relatively high exposure time used in LEEM (few hundred milliseconds). Island 3 in Figure 1g, however, captured in two different locations (3 and 3′), allows to make a lower estimate of the diffusion velocity, *v*_*d*_ = 700 nm s^−1^. Alternative estimations exploiting the sometimes smeared appearance of the islands (see Section S2, Supporting Information) suggest *v*_*d*_ = 790 − 1900 nm s^−1^.

## 3 Registry Index Simulations

**Figure 2:**
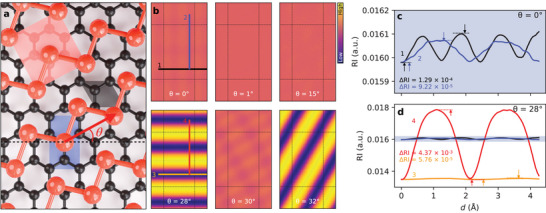
Registry index simulations. a) Ball‐and‐stick model of the α‐Bi (red)/graphite (black), with unit‐cells shown by semi‐transparent regions of the same colors. Only the atomic layers at the interface are shown. The blue area highlights to the translation space of RI maps. The zigzag direction of graphite (dashed line) and α‐Bi's $R_{1}^{\prime }$ (red arrow) are indicated. b) RI maps for selected twist angles specified on the RI maps. Dashed lines correspond to borders of the translation space. c,d) RI profiles taken from solid lines of the same colors in (b), for θ = 0° (c) and θ = 28° (d) (dotted lines and arrows indicate minima and maxima). The shaded areas in (c,d) illustrate the difference in absolute RI values across the different example paths. The profiles in (b–d) are also numbered for clarity.

As evidenced by LEEM, the trajectories of the α‐Bi islands are rotated by 60°, suggesting that the hopping tracks follow crystallographic directions. The observed behavior suggests a partly commensurate superlubricity, where the α‐Bi adsorbate layer forms a 1D superlattice. This remains in contrast to most commensurate systems defined with two non‐colinear commensurate vectors.^[^ ^13^ ^]^ The µ‐LEED image in Figure 1b shows that the reciprocal lattice vectors **Bi**(12) and **G**(01) are superposed (and correspond to a unique coincidence vector), as expected for type‐B contact leading to directional superlubricity. **Figure** **2**a shows the crystal structures of α‐Bi and HOPG at the interface (deeper graphene monolayers, and α‐Bi above the first atomic layer in contact with graphite are ignored).

To gain insight into the nanoscale friction properties of α‐Bi/HOPG, we perform RI simulations on finite 2D α‐Bi flakes for different twist angles. The RI is the overlap area between the substrate and the adsorbate interfacial lattices, using circular domains for each atomic site.^[^ ^3^ ^]^ RI maps, obtained by calculating the RI for a range of lateral translation vectors **r**_*C*_, can approximate *U*(**r**_*C*_) of the adsorbed layer^[^ ^3^, ^4^ ^]^ (more details on the simulations are given in Section S4, Supporting Information). Figure 2b shows RI maps for an α‐Bi slab comprising of 30 × 30 unit‐cells (1800 atoms) for several twist angles. For several twist values (θ = 0, 1, 15 and 30°), the potential energy landscape is nearly uniform, meaning that the diffusion barrier is independent of the direction. In contrast, the RI maps obtained for θ = 28° and θ = 32° possess a strong 1D character, consistent with large friction anisotropy. The global RI minima are found for these two twist angles suggesting these are the most energetically favorable configurations (see RI maps obtained for a large range of twist angle in Figure S1, Supporting Information), in agreement with the observed twist angles in the experiment. The two special twist angles correspond to the two symmetrically equivalent partly commensurate configurations, **Bi**(12) = **G**(01) and $\mathrm{Bi}\left(\bar{1}2\right)=G\left(\bar{1}1\right)$ respectively (see Section S1 for additional information, Supporting Information), corresponding to the energy landscapes of islands 3 and 4 above, consistently with their minor difference in twist. The low RI pathways correspond to the *nanohighways*, demonstrated for microscale colloidal particles.^[^ ^13^ ^]^

To further characterize the potential energy barriers associated with the translation of α‐Bi, we consider line profiles across the RI maps to qualitatively compare frictional properties for different twist angles and different hopping directions. Several profiles visible as color lines in Figure 2b are shown in Figure 2c,d. The RI profiles along graphite's zigzag (black) and armchair (blue) directions are very similar, showing a maximum corrugation of ΔRI = 1.29 × 10^−4^ and ΔRI = 9.22 × 10^−5^ respectively (a ratio of ≃ 1.4) consistent with isotropic behavior. In contrast, the equivalent RI profiles along the same translation directions for θ = 28° are characterized with significantly different energy barriers (ΔRI = 5.76 × 10^−5^ and ΔRI = 4.73 × 10^−3^ for profiles along graphite's zigzag and armchair translation, respectively) with a ratio ≃ 76, consistent with anisotropic friction behavior. RI profiles 1 and 2 in Figure 2c show corrugations comparable to profile 3 in Figure 2d, however, 1 and 2 correspond to a larger absolute RI value, indicating that profile 3 is energetically favorable. The RI corrugation along the preferential directions is substantially vanished and in fact decreases with the α‐Bi slab size (see Section S4 and Figure S4 for size‐dependent simulations, Supporting Information). These unique translational energy potential landscapes are in agreement with theory for type‐B contacts.^[^ ^13^ ^]^

## 4 Hopping Statistics

**Figure 3:**
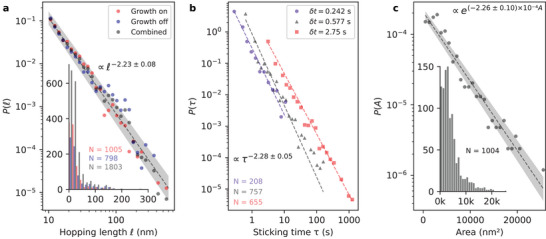
Statistics of hopping lengths and sticking times. a) Log–log histogram of hopping lengths ℓ with (red), without (blue) incoming Bi flux and combined (grey). b) Log–log histogram of sticking times τ for different frame rates δ*t* = 0.242 s (blue circles), 0.577 s (black triangles) and 2.75 s (red squares). c) Semi‐log histogram of areas during an hopping event. *N* is the number of events. The shaded areas correspond to the uncertainty in the lines of best fit. The numerical expressions resulting from fitting in (a, b) specified directly in the panels are extracted from the most populated series (“combined” and δ*t* = 0.577 s, respectively). All values of η_ℓ_ and η_τ_ are specified in the text. Insets in (a, c) show the same data in absolute counts on a linear scale.

We now focus on the statistical behavior of active α‐Bi islands observed by LEEM by measuring the hopping length ℓ=|r(t+δt)−r(t)| (where δ*t* is the time step in LEEM sequences). **Figure** **3**a shows the distribution *P*(ℓ) with (growth on) and without incoming Bi flux (growth off). The range is limited by pixel size (δ*x* = 10 nm, hopping events such that ℓ < 10 nm are discarded from analysis). The large majority of diffusion events are characterized with a small hopping length (80% of the observed hopping events are below 40 nm), while the longest is just under 600 nm. Surprisingly, *P*(ℓ) agrees with a power law distribution $P\left(\ell \right)∼\ell^{-\eta_{\ell }}$ with $\eta_{\ell }=2.23\pm 0.08$ (see dashed line) independently of the Bi flux ($\eta_{\ell }^{\mathrm{on}}=2.34\pm 0.09$ and $\eta_{\ell }^{\mathrm{off}}=2.12\pm 0.11$, best‐fit lines hidden for clarity). The distributions *P*(ℓ) of active individual islands are shown in Figure S6 (in Section S5, Supporting Information) and evidence the heavy‐tailed nature of the hopping length distribution at the individual island level with very similar decay parameters (the majority of islands have η_ℓ_ ≃ 2.4). Note that the decay parameters η are determined using maximum likelihood estimation.^[^ ^51^, ^52^ ^]^

Besides the hopping lengths, the duration τ during which an island remains immobilized between successive hopping events, or *sticking time*,^[^ ^53^ ^]^ is also monitored. The distribution of the sticking times *P*(τ) is shown in Figure 3b. Here, the range is limited by the frame rate δ*t* which sets a lower bound on the observable, i.e., τ ≥ δ*t*. If an island is observed in two distinct locations in successive LEEM frames, then τ = δ*t*; if not, τ is integrated until the next hopping event is observed. *P*(τ) also agrees with a power law $\tau^{-\eta_{\tau }}$ with η_τ_ = 2.28 ± 0.05 (for the largest population, with δ*t* = 0.577 s), remarkably similar with the decay parameter in the hopping lengths distributions *P*(ℓ) in Figure 3a. Distributions obtained for different frame rates (δ*t* = 0.242 s and 2.75 s) also show similar values, η_τ_ = 2.12 ± 0.08 and η_τ_ = 1.93 ± 0.04 respectively. The pause in the hopping activity of island 1 for about 100 s, described earlier, is in agreement with the stochastic nature of the hopping. Longer sticking times are occasionally observed, up to τ ∼ 1000 s as shown in Figure 3b.

The areas *A* of all active islands are extracted at every hopping event during the LEEM sequence. The histogram in Figure 3c shows that large islands are less likely to hop, although islands as large as 20 000 nm^2^ show occasional hopping. As opposed to hopping lengths ℓ and sticking times τ, the distribution of areas *P*(*A*) is described by an exponential distribution *P*(*A*) ∼ exp (− α · *A*). For an ideal structural lubrication system, the frictional properties are independent of the system size in contradiction to our observations. While we cannot rule out edge pinning effects^[^ ^11^ ^]^ (which may be further complicated here because α‐Bi undergoes edge reconstruction^[^ ^54^ ^]^), this size‐dependence is attributed instead to the enhanced pinning probability of large islands due to the broader contact area at the interface. In fact, we show (see Section S6, Supporting Information) that an exponential dependence is in agreement with a model where randomly‐distributed point defects in the substrate's surface are responsible for island pinning. The exponential decay parameter α = (2.26 ± 0.10) × 10^−4^ nm^−2^ is directly related to the density of graphite point defects in the middle of terraces (excluding the strong pinning of islands decorating terrace step edges); under such model this implies a defect density of ρ = (2.32 ± 0.11) × 10^10^ cm^−2^, in rough ballpark with high‐quality HOPG.^[^ ^55^ ^]^ Additionally, large islands (at a late stage of deposition) are in closer proximity to one another and may coalesce and form grain boundaries,^[^ ^19^ ^]^ likely to promote pinning and to suppress structural superlubricity. Despite a surprising numerical similarity with η_ℓ_ and η_τ_, α has a unit of surface density characterizing *P*(*A*), and we are therefore cautious about establishing a correlation between power‐law and exponential coefficients in these different statistical distributions.

## 5 Discussion, Outlook, and Conclusion

### 5.1 Discussion

Most random walk processes are governed by exponential distributions, such as Brownian motion.^[^ ^53^, ^56^ ^]^ Adsorbed gold clusters and single metallic adatoms on graphene show heavy‐tailed hopping distributions in molecular dynamics simulations^[^ ^53^, ^57^ ^]^ as well as a limited number of experimental systems at liquid‐solid interfaces observed by TEM.^[^ ^47^, ^58^ ^]^ In principle, the hopping α‐Bi islands could be observed via TEM (or other microscopy techniques), however this would require using an in‐situ apparatus as Bi oxidizes readily in ambient atmosphere.^[^ ^19^, ^20^ ^]^ In our experiment the flight durations are unfortunately inaccessible due to the low frame rate in LEEM. Under the hypothesis that the velocities of the islands during a diffusion event are narrowly distributed, it follows that the distribution of hopping durations *P*(*t*) should agree with a power law similar to that of *P*(ℓ). Nonetheless, the sticking time distribution *P*(τ) in our data agrees with that of a system whose dynamics are identified as Lévy flights.^[^ ^53^ ^]^ Heavy‐tailed distributions^[^ ^59^ ^]^ can be called Lévy flights when both diffusion lengths ℓ and durations *t* follow power‐law distributions with similar exponents η_*t*_ ≃ η_ℓ_ > 1. These scaling exponents describe how rare the large hopping events or large sticking times are, in comparison with hopping events of shorter lengths that are more frequently observed. These parameters reveal a fundamental heavy‐tail nature in the hopping characteristics of the Bi islands on HOPG and are independent of the experimental conditions. Previously described in animal colonies^[^ ^60^, ^61^, ^62^ ^]^ (wherein the heavy tail in flight lengths is crucial to the optimization of foraging patterns), these statistics describing random walks are also present in a multitude of other complex systems such as financial,^[^ ^63^, ^64^ ^]^ geophysical^[^ ^52^, ^65^, ^66^ ^]^ and photonic.^[^ ^67^, ^68^ ^]^ Lévy flights in solid‐state diffusion have however remained elusive, with rare published realizations^[^ ^35^ ^]^ phenomenologically distinct from our work, e.g., particles trapped in vortices,^[^ ^69^ ^]^ polymer aggregates in solution,^[^ ^70^ ^]^ or single Pd adatoms on W(211) substrates^[^ ^71^ ^]^ diffusing over angstrom‐length scales, in contrast to the many orders of magnitudes in hopping lengths (and mass scales) reported here. The connexion between the directional superlubricity and Lévy flights remains to be elucidated, although we suspect that the 1D confinement of the islands along *nanohighways* might enhance long‐distance hopping. We hope our results provide a basis for future theoretical investigations on these effects.

### 5.2 Outlook

Expanding the size of the system realizing directional superlubricity would be a valuable direction of future research. We identify the reasons for the current size‐limitation as follows: (1) higher probability that the island covers a defect in the substrate, permanently pinning the island; (2) the presence of neighboring islands which can lead to coalescence, likely accompanied with the formation of grain boundaries^[^ ^19^ ^]^ therefore breaking the crystalline symmetry; (3) possible edge‐pinning effects,^[^ ^11^ ^]^ although we do not have evidence for this, and distinguishing edge‐driven and area‐driven effects are difficult. The theory of 1D Lévy flights in α‐Bi/graphite is not yet established, although it is clear that the distribution of graphitic superficial defects play a major role, in contrast to ref. [53] where the heavy‐tails in the simulated hopping lengths arise without the presence of defects. Despite this, the observed power‐law parameters η_ℓ_ and η_τ_ are in excellent agreement with gold clusters/graphite simulations.^[^ ^53^ ^]^ Future investigations employing ab‐initio and classical molecular dynamics simulations, in which the role of temperature, defect types and density (as well as morphological parameters such as island size, shape and edges) can be independently investigated, will certainly bring valuable insight. Additionally, nanotribological experiments involving the use of scanning probes which couple force and displacements may further characterize the α‐Bi/HOPG system in terms of frictional properties. Lastly, our results further evidence the crucial role of incommensurability in superlubricity, and we expect that other 2D van der Waals interfaces could also allow the observation of directional superlubricity. The vast family of layered van der Waals materials implies that many combinations of crystallographies are possible, each with different implications on the both the type of contact and the resulting frictional properties. Screening methods may prove particularly interesting to explore the types of incommensurate contacts, prior to lab testing.

### 5.3 Conclusion

Our LEEM experiments evidence spontaneous and directional diffusion of α‐Bi islands on graphite, explained by a type‐B structural superlubricity model identified recently.^[^ ^13^ ^]^ Our RI simulations agree very well with the observations and further evidence a unique directional superlubricity behavior, where two superlubric pathways (or *nanohighways*) separated by 60° are formed when α‐Bi islands are twisted by θ = 28° and 32°, which correspond to the observed twist angles in our samples. Finally, statistical analysis of both hopping lengths ℓ and sticking times τ reveal heavy‐tailed distributions (η ≃ 2.0 − 2.5 for both quantities) over many orders of magnitude, indicative of Lévy flight dynamics in the α‐Bi/HOPG system. We believe that these results should renew the search for Lévy flights in solid‐state physics and encourage further studies in the tribology of van der Waals contacts with distinct crystalline symmetries.

## 6 Experimental Section

### LEEM and LEED

The experimental data were obtained at the Elettra synchrotron in Trieste, Italy. HOPG (SPI‐1) substrates were cleaved in air before loading into the UHV chamber and degassed (*T* > 400 °C) for about 12 h. LEEM images were processed and analyzed using ImageJ for tracking; waterfall plots were obtained from cross‐sectional profiles across the images.

### Registry Index Simulations

Custom‐made script based on ref. [3] was developed for all RI simulations using python.

### Statistical analyses

Statistical analyses were obtained from the tracking data using Python. The decay constants (η_ℓ_, η_τ_ for the hopping lengths and sticking times, respectively) were obtained using maximum likelihood estimation,^[^ ^51^, ^52^ ^]^ and a standard least‐square method (scipy.optimize.curve_fit) using power law functional for the vertical offsets in log‐log plots. The exponential fitting of *P*(*A*) was performed using scipy.optimize.curve_fit.

## Conflict of Interest

The authors declare no conflict of interest.

## Author Contributions

M.L.S. prepared the manuscript and performed the RI simulations, P.J.K., A.L., T.O.M. and S.A.B. conducted the experiment at the Elettra synchrotron, P.J.K. and K.P. performed the image analysis and statistical analyses. All authors contributed to the discussion equally. The authors declare no competing interests. The data is available upon reasonable request to the corresponding author.

## Supporting information

Supporting Information

Supplemental Video 1

Supplemental Video 1

Supplemental Video 1

## Data Availability

The data that support the findings of this study are available in the supplementary material of this article.
