# Supplementary material for: Evidence of Directional Structural Superlubricity and Lévy Flights in a van der Waals Heterostructure
Source: Small. 2024 Nov 26;21(6):2408349. doi: 10.1002/smll.202408349 (PMC11817913; doi:10.1002/smll.202408349)
Supplement: Supplementary file 1 — Supporting Information [file SMLL-21-2408349-s004.pdf]

NANO · MICRO
small

Supporting Information

for *Small*, DOI 10.1002/smll.202408349

Evidence of Directional Structural Superlubricity and Lévy Flights in a van der Waals Heterostructure

Maxime Le Ster, Paweł Krukowski, Maciej Rogala, Paweł Dabrowski, Iaroslav Lutsyk, Klaudia Toczek, Krzysztof Podlaski, Tefvik Onur Menteş, Francesca Genuzio, Andrea Locatelli, Guang Bian, Tai-Chang Chiang, Simon A. Brown and Paweł J. Kowalczyk**

Supplementary Information

Evidence of Directional Structural Superlubricity and Lévy Flights in a van der Waals Heterostructure

M. Le Ster *et al.*

S1. COINCIDENCE VECTORS

Figure S1(a) shows the reciprocal lattices of α-Bi (red) and graphite (black) for $\theta = 30°$, similar to the observed values of the twist angles. It is clear that $\mathbf{Bi}(\bar{1}2)$ and $\mathbf{G}(\bar{1}1)$ and $\mathbf{Bi}(12)$ and $\mathbf{G}(01)$ are in close proximity in reciprocal space and are therefore (independently) candidates for type-B commensurability [1]. We define the two lattices such that either $\mathbf{Bi}(\bar{1}\bar{2})$ and $\mathbf{G}(\bar{1}1)$, or $\mathbf{Bi}(\bar{1}2)$ and $\mathbf{G}(01)$ are superposed. The two distinct coincidence conditions are:

$$\mathbf{Bi}(\bar{1}2)(\theta_1) = \mathbf{G}(\bar{1}1) \tag{1}$$

$$\mathbf{Bi}(12)(\theta_2) = \mathbf{G}(01). \tag{2}$$

We develop eqs. (1, 2) to obtain θ_1 and θ_2. With the 2D unit cells defined as in our previous work [2] with $\mathbf{R}_1, \mathbf{R}_2$ vectors (with lengths r_1, r_2, respectively) spanning the real space unit cell and separated by an angle ω (ω referring to the substrate, here graphite's $\omega = 120°$, and α-Bi's $\omega' = 90°$). Conditions (1) and (2) imply a lattice parameter condition (here defined in reciprocal space) as follows:

$$\begin{cases} ||\mathbf{Bi}(\bar{1}2)|| = ||\mathbf{G}(\bar{1}1)|| \\ ||\mathbf{Bi}(12)|| = ||\mathbf{G}(01)||. \end{cases} \tag{3}$$

By symmetry, the two equations in eq. (3) are identical. Either can be independently developed into the condition dictating the real space lattice parameters r'_1 and r'_2 of α-Bi as follows:

$$r'_2 = \frac{1}{\sqrt{\frac{1}{3a^2} - \frac{1}{4r'^2_1}}} \tag{4}$$

valid for $r'_1 > \frac{a\sqrt{3}}{2} = 2.13...$ ($r'_1 \simeq 4.5$ Å, so eq. (4) has a valid domain of applicability). Figure S1(b) shows the set of lattice parameters (r'_1, r'_2) such that α-Bi/graphite is commensurate (for both conditions in eqs. (1, 2)) using $a = 2.461$ Å. Additionally, a twist angle

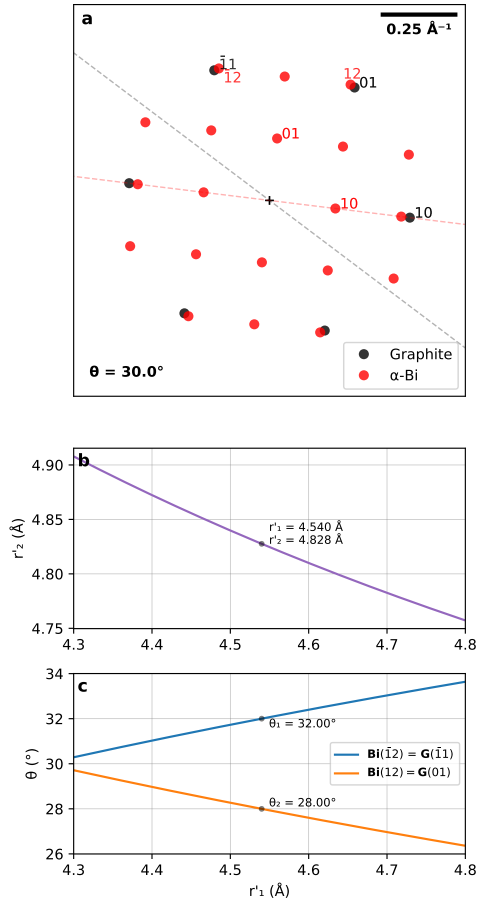

FIG. S1. **One-dimensional commensurate condition in reciprocal space.** (a) Reciprocal lattices of α-Bi (red) and graphite (black) for $\theta = 30°$. The dashed lines correspond to the zigzag directions of both crystals (separated by θ). The reciprocal lattice points pairs ($\mathbf{Bi}(\bar{1}2)$, $\mathbf{G}(\bar{1}1)$) and ($\mathbf{Bi}(12)$, $\mathbf{G}(01)$) are in close proximity. (b) α-Bi's lattice parameters (r_1, r_2) satisfying both $\mathbf{Bi}(\bar{1}2) = \mathbf{G}(\bar{1}1)$ and $\mathbf{Bi}(12) = \mathbf{G}(01)$, which are equivalent by symmetry. (c) Twist angle θ_1 and θ_2 satisfying both commensurate definitions as a function of r'_1.

condition imposed by commensurability conditions in eqs. (1, 2) must be considered, which are determined by basic trigonometry. The resulting twist angles θ as a function of r'_1 are shown in Fig. S1(c). The two twist values $\theta_1 = 32.00°$ and $\theta_2 = 28.00°$ are in very good agreement with the experimental observations in the main paper. Bismuthene's lattice parameters can be modified slightly whilst maintaining the commensurate conditions (1) or (2) as shown in Fig. S1(b, c).

Two other reciprocal lattice points are in relative proximity: $\mathbf{Bi}(20)$ and $\mathbf{G}(10)$. Un-

der sufficient uniaxial compressive strain of the α-Bi layer ($\varepsilon_1 = -6.1\%$) the 1D commensurate condition can be met for $\theta = 30°$, which is likely to imply $\mathbf{Bi}(12) = \mathbf{G}(01)$ and $\mathbf{Bi}(\bar{1}2) = \mathbf{G}(\bar{1}1)$, in this case satisfied with an additional tensile strain along the perpendicular direction $\varepsilon_2 = +1.9\%$. Such 2D commensurate condition does not correspond to the lattice parameters of α-Bi on graphite and disagrees with our μ-LEED experiments, and would correspond to a standard commensurate type-A contact, prohibiting superlubricity. The presence of an easy hopping direction in the experiments allows to discard such epitaxial relationship.

To summarize, there are two commensurate cases for α-Bi/graphite in the vicinity of $\theta = 30°$ for a fixed rigid lattice (4.540×4.828 Å^2) minimizing strain with respect to accepted lattice constant values [2–7], *i.e.*, for $\theta_1 = 32°$ and $\theta_2 = 28°$ which correspond to $\mathbf{Bi}(\bar{1}2) = \mathbf{G}(\bar{1}1)$ and $\mathbf{Bi}(12) = \mathbf{G}(01)$, respectively. A single pair of reciprocal lattice points from bismuthene and graphite can be superimposed at once ($\Omega = \{\mathbf{Bi}(12)\}$ or $\Omega = \{\mathbf{Bi}(\bar{1}2)\}$), indicative of a type-B contact [1].

S2. DIFFUSION VELOCITY

The LEEM experiments allow an estimate of the diffusion velocity of α-Bi islands on HOPG. Three approaches to estimate the diffusion velocity v_d are explained below.

a. Case 1. Island 3 in the main text in Fig. 1(g) is captured in two distinct locations. This indicates that the diffusion velocity is much faster than the velocity limit imposed by the LEEM frame rate ($\delta t = 2.75$ s). The LEEM image shows that the island has to be in location 3 and 3′ within the shorter span of the acquisition time ($\delta t_{\mathrm{acq}} = 350$ ms), during which the island is recorded in the two separate positions ($\Delta x \simeq 100$ nm) for 30-40% of the acquisition time, *i.e.*, 140 ms. This allows for an estimate of the lower bound of the diffusion velocity, approximately $v_d = 700$ nm/s.

b. Case 2. Fig. S2 shows four consecutive LEEM images showing the diffusion of a single α-Bi island. The island diffuses from the top of the image in Fig. S2(a) by about 100 nm lower in the next LEEM image in Fig. S2(b). The island in Fig. S2(c) is smeared indicating that it was captured during a diffusion event. Finally, Fig. S2(d) shows the island in its final position, approximately 550 nm away from the initial position in Fig. S2(a). The smearing distance ($\Delta x \simeq 150$ nm) and the acquisition time ($\delta t_{\mathrm{acq}} = 150$ ms) allow to make

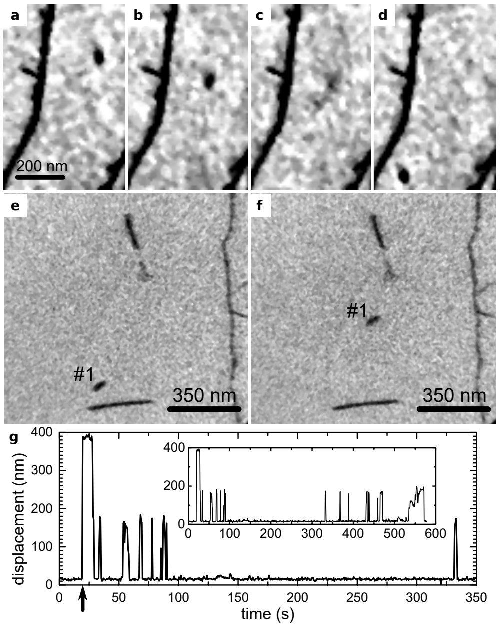

FIG. S2. **Estimating the diffusion velocity.** (a-d) Four consecutive LEEM images showing the diffusion of a single α-Bi island. (e, f) Two consecutive LEEM frames (in a different sequence) showing rapid movement of the island labelled #1. (g) Trajectory of island #1 extracted for a 350 s duration (inset: trajectory over a 600 s period). The arrow indicates the time at which frames (e) and (f) are obtained. See MOV-2 for the LEEM sequence corresponding to (e-g).

another estimate with $v_d = 1000$ nm/s.

c. Case 3. Fig. S2(e) shows an active island (labelled island #1) visible at the bottom of the image, and is observed at a different location in the following frame in Fig. S2(f). The displacement of island #1 shown in Fig. S2(g) reveals a hopping length of $\ell = 360$ nm ($\delta t = 430$ ms and $\delta t_{\text{acq}} = 250$ ms). Note that the island does not show any smearing and its location is well defined, meaning that it was stationary during the acquisition in both images and as a consequence diffused during a maximum duration of 180 ms across the two locations, allowing for an estimate $v_d = 1900$ nm/s.

S3. THERMAL ACTIVATION

Our observations show that in the initial stages of deposition, α-Bi islands are more active and roughly $\sim 20\%$ of them show spontaneous anomalous diffusion. However after some time, the islands typically reach a steady state and the hopping activity decreases, or

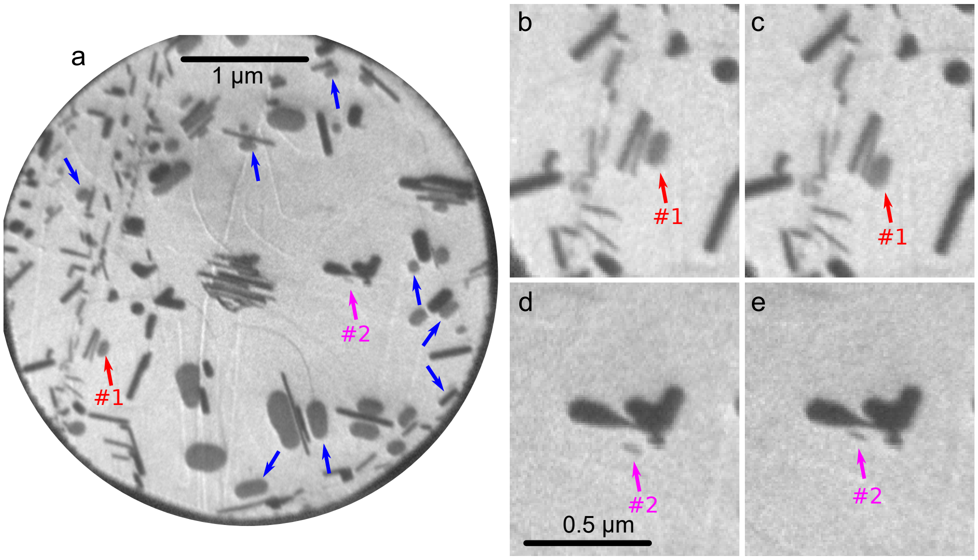

FIG. S3. **Thermal reactivation.** (a) Last frame of the LEEM sequence recorded at approximately 450 K (see MOV-3 for the corresponding LEEM movie). Arrows indicate several islands that become active after increasing the temperature. The red and pink arrows highlight islands #1 and #2 that are reactivated, shown in higher magnification in consecutive frames (b,c) and (d,e) respectively.

vanishes completely. In order to verify whether it is possible to thermally reactivate these islands, we perform a LEEM acquisition with a mild increase of temperature, from room temperature (~ 300 K) to $400-450$ K. Figure S3(a) shows a LEEM image where several islands undergo an unpinning process by thermal activation.

S4. RI SIMULATIONS

Registry index (RI) simulations [8] (see Fig. 2(b) in the main text) allow to analyze the interaction between two crystalline bodies in contact in terms of relative twist and lateral translation. The RI is equal to the sum of the overlapping area between circles (representing atoms at the crystalline interface). The circles have a radius $r = 0.3a$ where a is the bond length ($a_G = 1.42$ Å, $a_{Bi} = 3.05$ Å).

The RI calculations were performed using different α-Bi slab sizes (5×5, 10×10, 20×20 and 30×30) where $r_1' \times r_2' = (4.540 \times 4.828)$ Å^2 on a graphite lattice ($r_1 = r_2 = 2.461$ Å). Refer to ball-and-stick models in Fig. 2(a) in the main text. Figure S4 shows RI maps

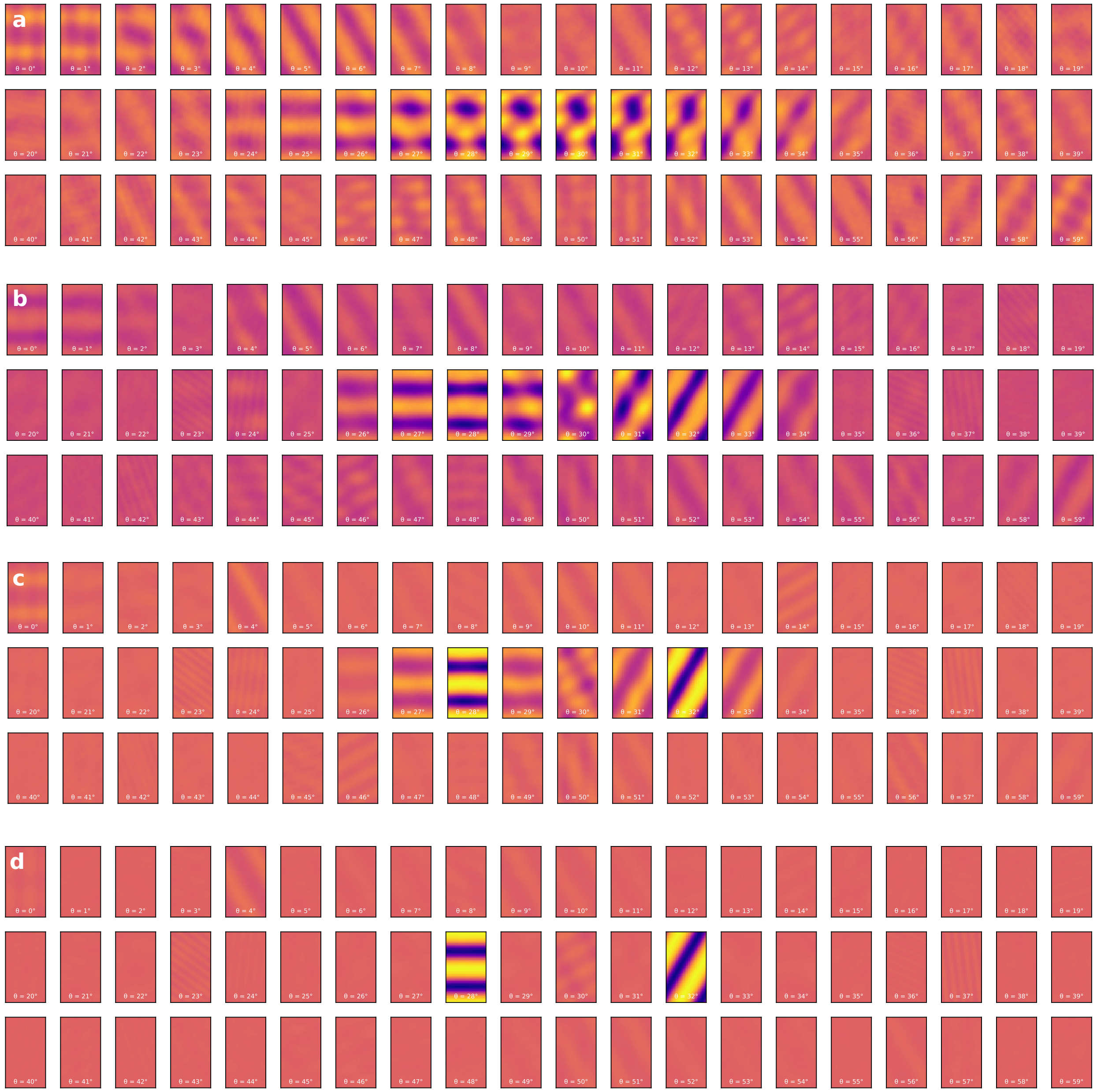

FIG. S4. **RI maps for different slab sizes.** RI maps of α-Bi/HOPG for a varying twist angle $\theta = 0°$ to $\theta = 59°$ ($\Delta\theta = 1°$) obtained with different α-Bi slab sizes: (a) 5×5 ($N = 50$ atoms), (b) 10×10 ($N = 200$ atoms), (c) 20×20 ($N = 800$) and (d) 30×30 ($N = 1200$ atoms).

obtained for a large range of twist angles ($\theta = 0$ to $\theta = 59°$ with $\Delta\theta = 1°$), for three different α-Bi slab sizes: 5×5, 10×10 and 30×30. For the smallest slab size in Fig. S4(a) the interlocking potential is minimized for $\theta \sim 30°$, however the RI corrugation for twist angles in this region are not unidirectional. The unidirectional character of the RI maps for these twist angles become visible for larger slabs (10×10, see Fig. S4(b)), and is very

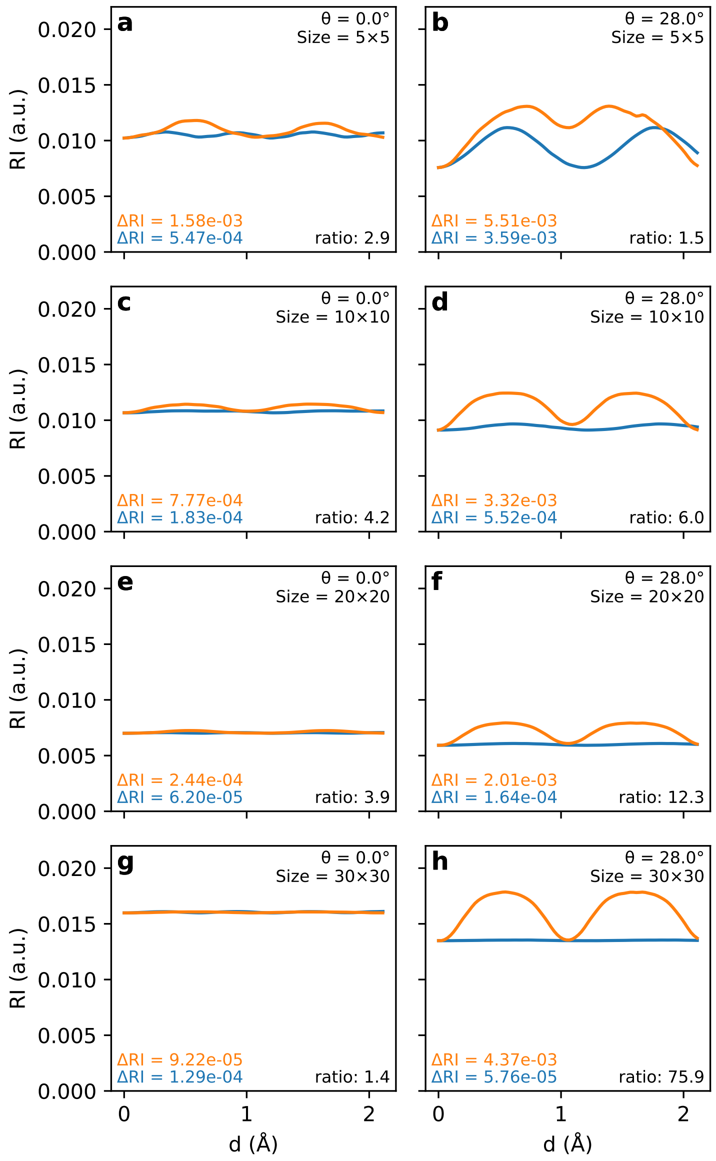

FIG. S5. **RI profiles for different slab sizes.** RI profiles across RI maps obtained for (a,b) (5×5), (c,d) (10×10), (e,f) (20×20) and (g,th) (30×30) α-Bi slabs, for twist angles (a, c, e, g) $\theta = 0°$ and (b, d, f, h) $\theta = 28°$. The profiles are obtained along the graphite's zigzag (blue) and armchair (orange) directions (line profiles shown in Fig. S4).

pronounced for the 30×30 slab as shown in Fig. S4(d). This behaviour, where the potential energy landscape becomes more unidirectional with the adsorbate layer size, is in agreement with the theory [1].

Fig. S5 shows RI profiles obtained horizontally (graphite zigzag, blue) and vertically (graphite armchair, orange) across the RI maps shown in Fig. S4. The profiles are extracted

from $\theta = 0°$ (left panels) and $\theta = 28°$ (right panels). The maximum corrugation along the profiles, ΔRI are shown in all panels. Of particular importance are the blue plots for $\theta = 28°$ as they correspond to a measure of the translational energy landscape of α-Bi along the *nanohighway*. It is clear that the corrugation decreases with increasing the size of the slab. Furthermore the ratio $\Delta\text{RI}_{\text{armchair}}/\Delta\text{RI}_{\text{zigzag}}$ increases with the slab size, confirming the one-dimensional character of the low friction pathway. For twist angles characterized by full incommensurability such as $\theta = 0°$ in this case, the ΔRI ratio (indicative of the friction anisotropy) tends to decrease with the slab size (and therefore becoming 'more' isotropic), in agreement with fully incommensurate type-C contact [1].

In general, the size-dependent simulations confirm the trend by which the one-dimensional character of the energy landscape is emphasized as the α-Bi island size increases. These results agree with the theory [1], although it is important to keep in mind that the model considers rigid lattices and ignores lattice relaxation, which becomes significant for large sizes in the case in a multitude of van der Waals heterostructures.

S5. STATISTICAL TESTS

Statistical analyses were performed on a total of 56 active α-Bi islands from 9 movies containing over 8500 frames. Exponential fitting of area distributions (as shown in Fig. 3(c) in the main text) was performed via standard least-square methods; power-law fitting of hopping length and sticking time distributions (η and $\Delta\eta$, see Fig. 3(a,b) in the main text) was performed using maximum likelihood estimation [9, 10]. Due to pixel size, hopping lengths events $\ell < \Delta x = 10$ nm and areas $A < 100$ nm^2 are discarded from the analysis. The number of bins n in histograms is $n = \lfloor\sqrt{N}\rfloor$ with N the number of events.

Figure S6 shows the distributions of both hopping lengths ℓ and sticking times τ for individual islands. Taken individually, the hopping lengths also follow the same trend as the overall population (data shown in Fig. 3 in the main text), *i.e.*, $P(\ell) \sim \ell^{-\eta_\ell}$ and $P(\tau) \sim \tau^{-\eta_\tau}$ with $1.98 < \eta < 2.89$. The island-to-island variation is attributed to local inhomogeneities in the substrate, such as line or point defects, or due the presence of neighbouring islands in immediate proximity.

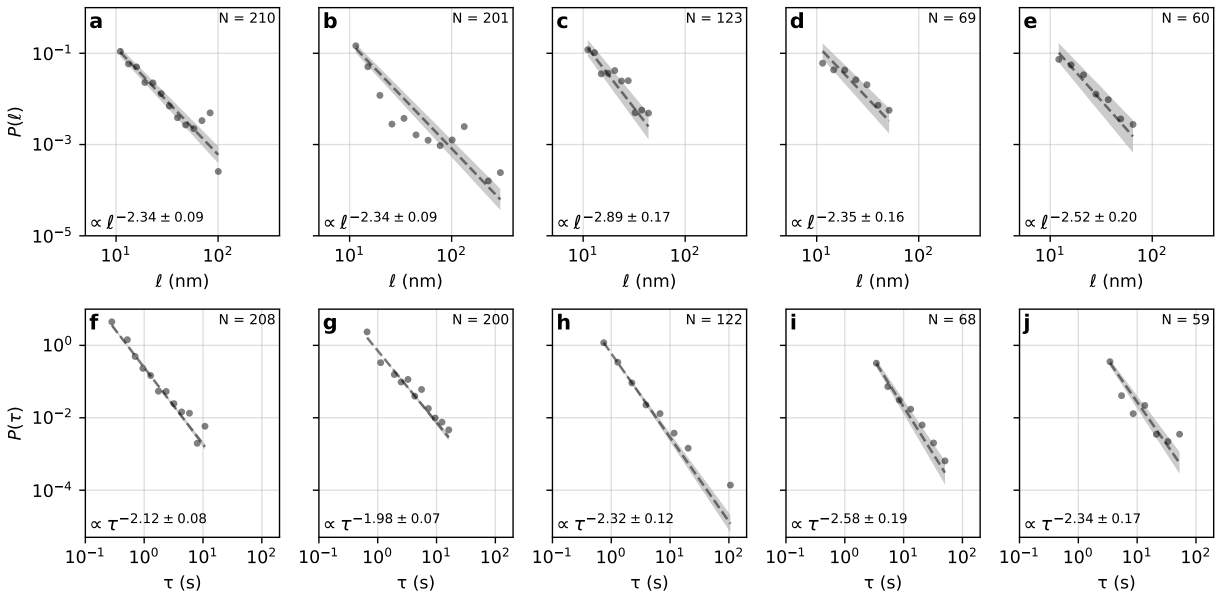

FIG. S6. **Statistics of hopping lengths and sticking times for individual islands.** Log-log histograms of (a-e) hopping lengths and (f-j) sticking times of individual islands with $N > 58$ (N number of events). Dashed lines correspond to power laws obtained via maximum likelihood estimation. The decay coefficients $\eta \pm \Delta\eta$ in of $P(\ell)$ and $P(\tau)$ are shown in the bottom left of each panel. The distributions are obtained from the same island in (a,f), in (b,g), in (c, h), etc.

S6. DEFECT DENSITY

In this section we investigate the role of defect density ρ on the hopping probability P, as a function of the island area A. We develop and employ a simple model to gain insight on statistical relationship between island area and defect density, which we describe as follows. We consider a surface populated with a number of point defects located at $\mathbf{r}_j$, determined by the defect density ρ. On top of the surface, we randomly distribute a number of islands of area A at location $\mathbf{R}_i$; for simplicity, we define the islands as circles. We fix the island density based on the experimental observations (excluding the islands decorating the terrace step edges, we estimate the island density $\varrho \simeq 1 \times 10^9$ cm^{-2}). Once the defects and islands are generated, we evaluate the number of islands N_{pin} that cover at least one point defect. This is achieved by evaluating the distance $d_{ij} = |\mathbf{R}_i - \mathbf{r}_j|$ between the island i and defect j, allowing to determine if the island i is pinned to defect j by testing $d_{ij} < r$ with r the radius of the circular island, $r = \sqrt{A/\pi}$. This model makes the assumption that the point defects

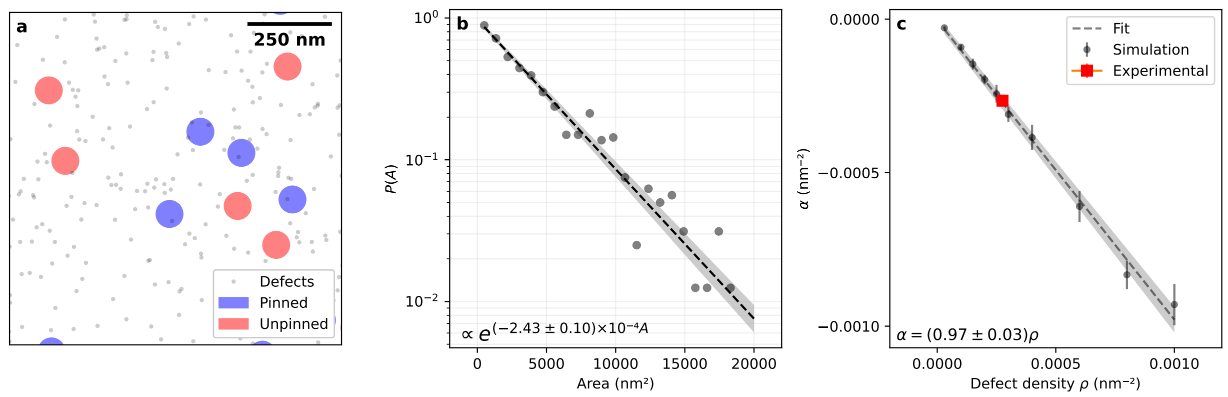

FIG. S7. **Substrate defect density and island pinning probability.** (a) Defect simulation example with defect density $\rho = 2.5 \times 10^{10}$ cm^{-2}, island density of $\varrho = 1.0 \times 10^9$ cm^{-2} and island area $A = 5000$ nm^2 (blue: pinned, red: unpinned islands). (b) Probability of an island to be unpinned as a function of the area $P(A)$, in agreement with an exponential decay probability density function (dashed line shows the line of best fit $\exp(-\alpha A)$ (c) Exponential parameter α dependence as a function of defect density in the simulations, in very good agreement with $\alpha = 0.97\rho \simeq \rho$. The red square corresponds to the experimental observation (shown in Fig. 3(c) in the main text), predicting a defect density $\rho_{exp} = (2.74 \pm 0.16) \times 10^{-4}$ nm^{-2}.

are responsible for island pinning, and thus, if an island covers at least one point defect in the simulation, the island is considered immobilized. The probability of island pinning $\bar{P}$ in that context is given by:

$$\bar{P} = \frac{N_{free}}{N_{total}} \tag{5}$$

with N_{free} the number of islands that do not overlap with a point defect ($N_{total} = N_{free} + N_{pin}$). The probability to find an active island (unpinned) is therefore given by:

$$P = 1 - \bar{P} = 1 - \frac{N_{free}}{N_{total}}. \tag{6}$$

The simulation consists of randomly positioning defects and islands based on their respective densities, and evaluating P as a function of the area of the island A (see Fig. S7(a) for schematics). Intuitively, large islands are more likely to overlap with a point defect; conversely small islands are more likely to sit between defect sites promoting higher hopping activity; therefore $P(A)$ is a decreasing function. The simulation is run for a 4×4 μm^2 surface (*i.e.*, for $N_{total} = 160$ islands), with areas A varying from 500 up to 20000 nm^2, as observed in our experiments.

Figure S7(b) shows the probability $P(A)$ for an example defect density of $\rho = 2.5 \times 10^{10}$ cm^{-2}, which agrees very well with $P(A) \sim \exp(-\alpha A)$ with $\alpha = (2.43 \pm 0.10) \times 10^{-4}$ nm^{-2}. Interestingly, the simulated probabilities are in very good agreement with the hopping probability $P(A)$ shown in Fig. 3(c) in the main text. For completeness, we run a series of similar simulations (with identical island density ϱ) this time with varying defect densities ρ ranging from 3×10^{-5} to 1×10^{-3} nm^{-2} (not shown). Figure S7(c) shows the resulting decay coefficients α as a function of the defect density ρ. Interestingly, the defect density and the decay coefficient follow an identity rule (with a minus sign prefactor) with an excellent agreement, $\alpha/\rho = 0.97 \pm 0.03$. This allows to calculate the point defect density responsible for large island pinning, $\rho_{exp} = (2.32 \pm 0.11) \times 10^{10}$ cm^{-2}.

S7. LIST OF LEEM SEQUENCES

The following list details the LEEM sequences mentioned in the manuscript (accessible in supplementary information and/or by request to the corresponding authors).

- **MOV-1.** LEEM sequence ($\delta t = 2.75$ s) discussed in the main text and in Fig. 1 of the main text.
- **MOV-2.** LEEM sequence ($\delta t = 0.577$ s) discussed in section S2 and pictured in Fig. S2.
- **MOV-3.** LEEM sequence ($\delta t = 2.75$ s) recorded while increasing the temperature from room temperature to $\sim 400 - 450$ K. Discussed in section S3. Islands in the centre of the frame undergo ripening.

[1] Panizon, E. *et al.* Frictionless nanohighways on crystalline surfaces. *Nanoscale* **15**, 1299–1316 (2023).

[2] Le Ster, M., Maerkl, T., Kowalczyk, P. J. & Brown, S. A. Moiré patterns in van der Waals heterostructures. *Phys. Rev. B* **99**, 075422 (2019).

[3] Kowalczyk, P. J. *et al.* Electronic Size Effects in Three-Dimensional Nanostructures. *Nano Lett.* **13**, 43–47 (2013).

[4] Kowalczyk, P. J. *et al.* Origin of the moiré pattern in thin Bi films deposited on HOPG. *Phys. Rev. B* **91**, 045434 (2015).

[5] Kowalczyk, P. J. *et al.* Realization of Symmetry Enforced Two-Dimensional Dirac Fermions in Nonsymmorphic α-Bismuthene. *ACS Nano* **14**, 1888–1894 (2020).

[6] Takahashi, K., Imamura, M., Yamamoto, I. & Azuma, J. Thickness dependent band structure of α-bismuthene grown on epitaxial graphene. *J. Phys. Condens. Matter* **34**, 235502 (2022).

[7] Bai, Y. *et al.* Doubled quantum spin Hall effect with high-spin Chern number in α-antimonene and α-bismuthene. *Phys. Rev. B* **105**, 195142 (2022).

[8] Hod, O. Interlayer commensurability and superlubricity in rigid layered materials. *Phys. Rev. B* **86**, 075444 (2012).

[9] Clauset, A., Shalizi, C. R. & Newman, M. E. J. Power-Law Distributions in Empirical Data. *SIAM Review* **51**, 661–703 (2009).

[10] Corral, Á. & González, Á. Power Law Size Distributions in Geoscience Revisited. *Earth Space Sci.* **6**, 673 (2019).
